# Wnt signalling in adenomas of familial adenomatous polyposis patients

**DOI:** 10.1038/sj.bjc.6605790

**Published:** 2010-07-13

**Authors:** G M Caldwell, C E Jones, A M Ashley, W Wei, R K Hejmadi, D G Morton, G M Matthews

**Affiliations:** 1 School of Cancer Sciences, The University of Birmingham, Edgbaston, Birmingham, B15 2TH, UK

**Keywords:** colorectal cancer, FAP, Wnt signalling

## Abstract

**Background:**

Epigenetic silencing of Wnt antagonists and expression changes in genes associated with Wnt response pathways occur in early sporadic colorectal tumourigenesis, indicating that tumour cells are more sensitive to Wnt growth factors and respond differently. In this study, we have investigated whether similar changes occur in key markers of the Wnt response pathways in the genetic form of the disease, familial adenomatous polyposis (FAP).

**Methods:**

We investigated epigenetic and expression changes using pyrosequencing and real-time RT-PCR in samples from seven patients without neoplasia, and matched normal and tumour tissues from 22 sporadic adenoma and 14 FAP patients.

**Results:**

We found that 17 out of 24 (71%) FAP adenomas were hypermethylated at *sFRP1*, compared with 20 out of 22 (91%) of sporadic cases. This was reflected at the level of *sFRP1* transcription, where 73% of FAP and 100% of sporadic cases were down-regulated. Increased expression levels of *c-myc* and *FZD3* were less common in FAP (35 and 46% respectively) than sporadic tumours (78 and 67% respectively).

**Conclusion:**

Overall, the changes in expression and methylation were comparable, although the degree of change was generally lower in the FAP adenomas. Molecular heterogeneity between multiple adenomas from individual FAP patients may reflect different developmental fates for these premalignant tumours.

Loss of *APC* gene activity is a common event in sporadic colorectal tumourigenesis, occurring in about 80% of cases (Morin *et al*, 1997). Germ-line mutation of the *APC* gene causes familial adenomatous polyposis (FAP) and this, along with mice carrying similar mutations, has been investigated extensively as a paradigm for sporadic disease (Fearnhead *et al*, 2001).

The majority of sporadic colorectal dysplasia and malignancy require inactivation of the *APC* gene, promoting *β*-catenin/TCF4-mediated transcription. Because *APC* functions within the Wnt/*β*-catenin arm of the Wnt signalling pathway, it may be expected that changes in these pathways and their actions associated with tumourigenesis would be similar in both genetic and sporadic diseases.

Previous studies have identified two further frequent Wnt pathway changes in sporadic tumours. First, secreted Wnt antagonists, both the sFRP family and the structurally unrelated WIF-1, are silenced epigenetically (Caldwell *et al*, 2004; Suzuki *et al*, 2004; He *et al*, 2005). These are *β*-catenin-responsive genes (Caldwell *et al*, 2006): in the absence of epigenetic silencing, *APC* mutation and consequent *β*-catenin action would lead to Wnt antagonist induction in a negative feedback loop (Caldwell *et al*, 2008), starving the tumour cell of Wnt ligand. Because Wnt deprivation of primary tumour cells in culture leads to apoptosis (He *et al*, 2005), this silencing may be necessary for survival of the early tumour.

The second Wnt-associated change observed in early sporadic tumours is a re-organisation of the Wnt response pathways away from *β*-catenin responses and towards the less well-characterised *β*-catenin-independent outcomes (Caldwell *et al*, 2008). These include changes in FZD receptor profiles and high-level induction of NKD1, which effectively diverts Wnt ligand-derived signals away from *β*-catenin and towards Wnt/PCP outcomes (Yan *et al*, 2001). In normal epithelia, these changes most likely represent transient negative feedback responses that desensitise the cell to high levels of Wnt ligands but their sustained expression in tumour cells may contribute to the tumourigenic process. Although the spectrum of outcomes has yet to be characterised, one result of Wnt/PCP signalling is c-jun phosphorylation, an essential step in colorectal tumourigenesis (Nateri *et al*, 2005).

Studies in FAP tissues and Apc^min^ mice could advance our understanding of the Wnt pathway changes observed in sporadic tumours. A number of studies using tissue from FAP patients have failed to show nuclear localisation of *β*-catenin in early adenomas with mild dysplasia, unlike sporadic adenomas (Anderson *et al*, 2002; Bläker *et al*, 2003; Phelps *et al*, 2009), which raises questions about the sequence of events following APC loss in FAP. Interestingly a recent article by Obrador-Hevia *et al* (2010) showed increased nuclear staining of *β*-catenin in FAP adenomas which correlated with larger size (adenomas >1 cm) and biallelic APC inactivation. Early changes in Wnt signalling pathways may provide targets for cancer prevention. A similar profile of changes must occur in FAP for it to be used as a model system to evaluate such therapies. Similarly, differences in changes in the Wnt signalling components between sporadic and genetic disease may further our understanding of the key mutational events in the development of this common tumour. We therefore set out to examine key components of the Wnt signalling pathways in FAP adenomas and compare any alterations with those found in sporadic cases.

## Materials and methods

### Patient samples

Sporadic adenoma samples were identified from our tumour bank from patients more than 50 years of age and without a family history of colorectal cancer. All tumour samples were collected from the sigmoid colon or the rectum and matched control rectal biopsies were taken at the same procedure (5 cm or more from the tumour site). Familial adenomatous polyposis adenoma and matched control samples were taken from patients with a proven diagnosis of FAP. Normal unaffected control samples were taken from patients with no gastrointestinal malignancy. A summary of the clinical characteristics of each sample group can be found in Table 1. Samples were collected according to local ethical committee regulations, with individual consent from each patient for molecular studies.

### Quantitative real-time RT-PCR

Total RNA was extracted from the patient samples using TRI Reagent (Sigma, Poole, UK) and 0.5 *μ*g total RNA was converted to cDNA using a High Capacity cDNA Reverse Transcription Kit (Applera, Warrington, UK). Oligonucleotide primers and TaqMan probe sequences are shown in Tables 1 and 2. Multiplex polymerase chain reaction (PCR) amplifications were performed using an ABI PRISM 7500 Sequence Detector (Warrington, Cheshire, UK) in a final volume of 15 *μ*l. Each reaction contained 1 × qPCR Mastermix (Eurogentec, Aylesbury, UK), 90 nM *KRT8* and target gene primers, 150 nM target gene TaqMan probe, and 175 nM KRT8 TaqMan probe and 1 *μ*l of cDNA. Cycling conditions were an initial step at 50°C for 2 min and 95°C for 10 min, followed by 40 cycles at 95°C for 15 s and 60°C for 1 min. Results from the human target genes were normalised to the epithelial-cell-specific gene keratin 8 (*KRT8*) (Table 3).

### Analysis of DNA methylation status using bisulphite/pyrosequencing

Bisulphite treatment of 1 *μ*g genomic DNA was carried out using the EpiTect Bisulfite Kit (Qiagen, Crawley, UK) following the manufacturer's recommendations. Primers adapted from the sFRP1 combined bisulphite restriction analysis (COBRA) assay previously described by our laboratory (Caldwell *et al*, 2004) were used to amplify 1 *μ*l of bisulphite-treated DNA. The primary PCR reaction product was diluted 100-fold and 1 *μ*l of this was added to a 50 *μ*l reaction containing 10 pmol COBRA-F primer and 5 pmol biotin-labelled COBRA-Rnest. Each reaction was amplified using a touchdown PCR; 94°C for 3 min; 20 cycles of 94°C for 10 s, 66–0.5°C for 20 s, 72°C for 30 s; 20 cycles of 94°C for 10 s, 56°C for 20 s, 72°C for 30 s and finally 72°C for 5 min. After PCR, the products were captured on streptavidin-coated beads and incubated with 10 pmol of sequencing primer (COBRA-Fnest). Pyrosequencing was performed using PyroMark Gold Q96 reagents and a PyroMark Q96 MD pyrosequencing machine (Qiagen). This assay measures the methylation status of seven CpG sites at positions +318, +321, +344, +351, +353, +357 and +370 relative to the *sFRP1* translation start site. CpG sites were hypermethylated if methylation was greater than 63%, this cut-off value corresponds to the average methylation in normal tissue +2 times the standard deviation (s.d.) as described previously (Chung *et al*, 2008).

### Immunohistochemistry

The streptavidin–biotin indirect immunoperoxidase method was performed as described previously (Hardy *et al*, 2002). Sections (5 *μ*m) were dewaxed and re-hydrated, then endogenous peroxidase activity was blocked by incubation with 10% H_2_O_2_ in methanol for 30 min. Microwave antigen-retrieval was undertaken for both antibodies. Sections were incubated overnight with a primary antibody recognising phospho-c-jun (ser63-P; Santa Cruz Biotechnology, Heidelberg, Germany) at a dilution of 1 : 400, diluted in 20% normal goat serum. After washing with PBS, sections were incubated with biotinylated goat anti-mouse/rabbit IgG (Dako, Ely, UK) according to the manufacturer's instructions for 30 min. Serial PBS washing and incubation with streptavidin–peroxidase conjugate (Dako) was undertaken before incubation with diaminobenzidine tetrahydrochloride (Sigma-Aldrich, Gillingham, UK). Sections were counterstained with haematoxylin (BDH Laboratory Supplies, Poole, UK), dehydrated and analysed on a light microscope.

### Statistical methods

Wilcoxon's signed-rank test was performed using R (http://www.r-project.org/). *P*-values <0.05 were considered as significant. Pearson's correlation coefficient (*r*) was calculated as a measure of the strength of the association between the two continuous variables. An *r* value >0.7 was considered statistically significant.

## Results

### Methylation status of sFRP1 promoter

**Figure 1:**
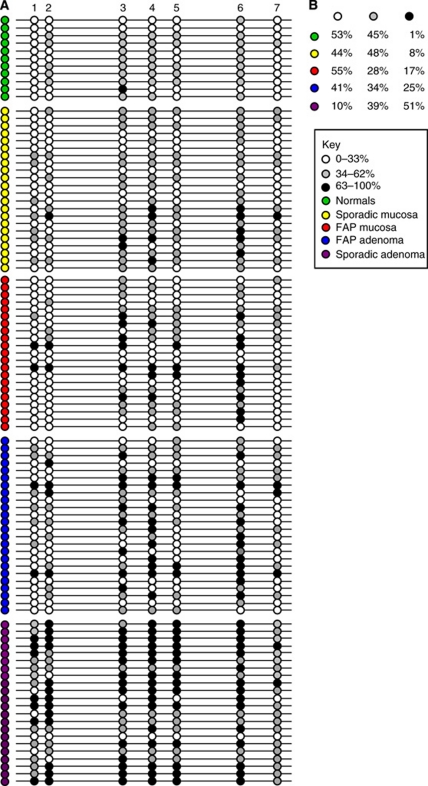
Bisulphite-pyrosequencing analysis of *sFRP1* gene methylation. (**A**) Pictorial representation of the percentage methylation of seven CpG sites within the first exon of the *sFRP1* gene. The average methylation in normal tissue was calculated as 33%, CpG sites with less than 33% methylation are shown as white circles. Fully methylated (above the cut-off) samples corresponded to average methylation in normal tissue + 2 times the standard deviation (s.d.) as described previously (Chung *et al*, 2008), these are shown as black circles (>63% methylation). Grey circles represent 34–62% methylation. Five sample groups are represented (normal unaffected samples (green), FAP normal mucosa (red), sporadic normal mucosa (yellow), FAP adenomas (blue) and sporadic adenomas (purple)). The FAP normal tissue shows an increase in methylation (more black circles) compared with unaffected and sporadic normal tissue. Both FAP and sporadic adenomas had increased methylation compared with the normal samples. (**B**) The table summarises the data in panel **A** as the percentage number of samples within each group with methylation 0–33% (white), 34–62% (grey) and >63% (black).

Our previous studies have shown hypermethylation of the *sFRP1* gene at the earliest stages of sporadic colorectal tumourigenesis (Caldwell *et al*, 2006). To determine whether hypermethylation of sFRP1 occurs in the large bowel mucosa and adenomas in FAP, we measured the methylation status of seven CpG sites close to the transcription start site of sFRP1. Samples from 14 patients with FAP (21 normal colon mucosa and 24 adenomas), 22 patients with sporadic adenomas (22 normal colon mucosa and 22 adenomas) and 7 patients without large bowel neoplasia (11 normal colon mucosa) were examined by bisulphite pyrosequencing. These results are summarised in Figure 1.

An average methylation score for each CpG site was calculated for the FAP patients where multiple control and adenoma samples were available (Table 2). Analysis of the data using the Wilcoxon's signed-rank test shows that *sFRP1* is significantly more methylated in adenoma compared with matched normal colonic mucosa in both FAP and sporadic patients (Table 4). The data show that while significant methylation changes occur at CpG sites 3 and 4 in FAP (*P*=0.03), they occur at all seven sites in sporadic disease (*P*=0.003).

Normal colonic mucosa from healthy controls and patients with sporadic disease was found to have a comparable degree of methylation, with only small differences observed between individual CpG sites (Figure 1). Only 1 out of 11 normal control samples showed methylation above 63% (black circle) and in only 1 out of the 7 CpG sites. In contrast, 10 out of 21 of the normal colon samples from FAP patients show methylation of 63% or greater at one or more CpG sites.

Adenoma samples from both FAP and sporadic patients showed increased methylation compared with matched normal mucosa. The sporadic adenomas showed the greatest degree of methylation. 90% of the CpG sites examined in sporadic adenomas were methylated compared with 59% in the FAP adenomas. This is particularly striking when examining the median methylation value for each CpG site (data not shown). The sporadic adenomas range from 51 to 70% methylation compared with a range of 25 to 61% methylation in the FAP adenomas. The data showed a greater level of methylation in FAP adenomas compared with matched normal mucosal biopsies at all CpG sites examined showing that methylation is a frequent and early event in FAP tumourigenesis although at a lower level than that observed in sporadic disease.

### mRNA expression profile of the Wnt antagonist *sFRP1*

**Figure 2:**
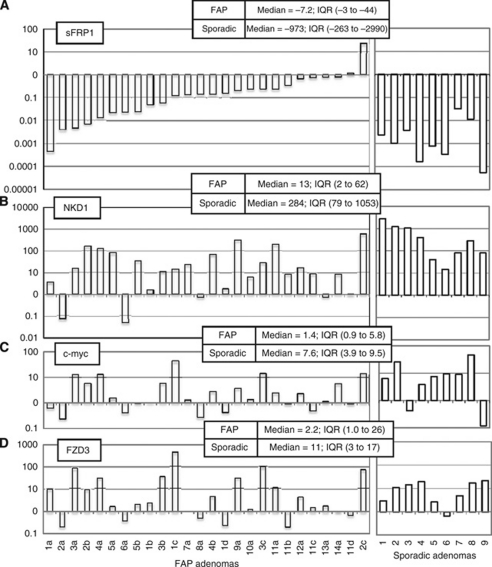
Real-time RT-PCR quantitation of relative mRNA expression in a series of 26 matched colorectal FAP tumours and 9 matched sporadic colorectal tumours according to the comparative *C*_T_ method as previously described (Caldwell *et al*, 2004). The inset shows the median and inter-quartile ranges (IQR) of expression levels for each gene in FAP/sporadic adenomas. **A**, *sFRP1*; **B**, *NKD1*; **C**, *c-myc*; **D**, *FZD3*. The sample numbers correspond to individual FAP patients and details of each adenoma can be found in Table 2.

Our previous studies identified loss of *sFRP1* expression at the premalignant stage of colorectal cancer development (Caldwell *et al*, 2006). To assess whether this is also seen in FAP-related tumourigenesis and whether the epigenetic changes identified in FAP were influencing expression, we measured mRNA levels using quantitative real-time RT-PCR. In total 26 adenomas taken from a cohort of 14 FAP patients aged between 18 and 38 years, and 9 sporadic adenomas taken from patients aged between 59 and 65 years were analysed. In each case the adenoma expression levels were compared with expression levels in normal bowel mucosa taken from the same patient as previously described (Caldwell *et al*, 2004). The results are summarised in Figure 2.

Both the FAP and sporadic adenomas showed a similar trend of down-regulation of *sFRP1* expression. Of 26, 19 (73%) FAP adenomas and all of the sporadic adenomas showed a greater than 4-fold down-regulation of *sFRP1*. The magnitude of change was different, with sporadic adenomas showing greater down-regulation (median –973-fold, IQR −263 to −2990) than the FAP adenomas (median −7.2-fold, IQR −3 to −44).

The widespread down-regulation of *sFRP1* in FAP cases corresponds with the epigenetic changes in sporadic adenomas in this and our previous study. The level of *sFRP1* methylation observed in FAP and sporadic adenomas reflects the degree of suppression of *sFRP1* expression, indicating it is causative. These data show epigenetic suppression of *sFRP1* to be a common and early event and are consistent with this being a requirement for the development of FAP and sporadic large bowel neoplasia.

### mRNA expression profile of *β*-catenin transcription target genes, *NKD1* and *c-myc*

To assess whether the *sFRP1* changes in FAP and sporadic colorectal adenomas reflected a modification of *β*-catenin transcriptional activity, we measured the expression levels of two *β*-catenin target genes, *NKD1* and *c-myc*, using quantitative real-time RT-PCR.

*NKD1* expression was up-regulated (>4-fold) in 19 out of 26 FAP adenomas and all 9 sporadic adenomas. The magnitude of up-regulation differed between the groups with sporadic adenomas showing nearly a 20-fold greater effect (Figure 2). A reduction in *NKD1* expression was observed in two FAP adenomas (2a and 6a) compared with their matched normal tissue.

*c-myc* was up-regulated (>4-fold) in 7 out of 9 (78%) sporadic adenomas and 9 out of 26 (35%) FAP adenomas. The nine FAP adenomas with increased *c-myc* also had increased levels of *NKD1*. In FAP sample 1a and 11c and sporadic samples 1 and 7, *c-myc* levels are reduced whereas *NKD1* is induced, suggesting other factors may be affecting *c-myc* expression.

### Evidence of Wnt pathway re-organisation in FAP adenomas

#### FZD3 induction

Our recent study (Caldwell *et al*, 2008) showed that Wnt response pathways are re-organised in early colorectal tumourigenesis: NKD1 induction is accompanied by up-regulation of the *β*-catenin-independent receptors FZD3 and 6. Unlike NKD1, however, FZD3/6 do not respond to stabilisation of *β*-catenin *in vitro* and require a Wnt ligand signal. FZD3/6 may, therefore, provide an indication of the degree of Wnt ligand signalling in the tumour.

We therefore measured *FZD* transcript levels in the two series of adenomas. Figure 2 shows that *FZD3* was induced by a factor of >4 in 6 of 9 sporadic adenomas and 12 of the 26 FAP specimens, suggesting a broadly similar incidence of Wnt response pathway re-organisation between the two groups.

The inter-quartile ranges of *FZD3* induction in the sporadic and FAP adenoma groups (3–17 *vs* 1.0–26) showed much greater similarity than a similar comparison for NKD1 (79–1053 *vs* 2–62).

An interesting observation in this study was the different expression profiles between different adenomas taken from the same FAP patient (see patients 1, 2 and 11, Figure 2). In sporadic adenomas the down-regulation of *sFRP1* correlates directly with an up-regulation of *NKD1*, but this is not always the case in the FAP adenomas. For instance in adenomas 2a and 6a *sFRP1* and *NKD1* are both down-regulated and in adenoma 8a *sFRP1* is down-regulated but there is no change in *NKD1* expression.

#### c-jun phosphorylation

**Figure 3:**
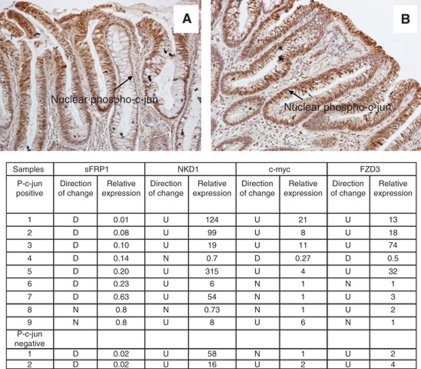
Immunohistochemistry results for phospho-c-jun in FAP adenoma sections. Panels **A** and **B** show the typical pattern of nuclear staining for phospho-c-jun in adenomas. The table summarises the mRNA expression data for each of the nuclear phospho-c-jun-positive and -negative samples (D, down-regulation; U, up-regulation; N, no change). The relative expression values are calculated by comparing gene expression in adenoma tissue with expression in matched normal mucosa.

We previously reported a correlation between Wnt response pathway re-organisation and phosphorylation of c-jun, an outcome of Wnt/PCP signalling, in sporadic adenomas (Caldwell *et al*, 2008). To investigate whether this occurs in FAP, we stained paraffin sections from 11 of the FAP patients to analyse for nuclear phosphorylated c-jun and examples of this are shown in Figure 3. Adenomas from these patients had also been analysed for mRNA expression of *β*-catenin transcription target genes (*sFRP1*, *NKD1* and *c-myc*) and the *FZD3* receptor.

Nuclear phospho-c-jun was observed in the majority, 9 out of 11 FAP adenomas. Fresh frozen tissue was available for mRNA analysis in the two FAP patient samples (patients 5 and 12), which stained negative for nuclear phospho-c-jun. Interestingly, patient 12 showed no change in *sFRP1* expression but did demonstrate induction of *NKD1*, *c-myc* and *FZD3*. Patient 5 showed a more typical molecular profile, that is, down-regulation of *sFRP1* and up-regulation of *NKD1* and *FZD3* although *c-myc* was unchanged. This suggests that in FAP, Wnt/PCP signalling is not the only factor involved in c-jun activation.

## Discussion

These data show considerable similarity between expression of Wnt components measured in premalignant sporadic and FAP large bowel adenomas. We have presented evidence to suggest the magnitude of these events may be greater in sporadic disease. These results are consistent with these changes involved in neoplastic development and/or stabilisation.

Age-related methylation of *sFRP1* has been reported (Konishi *et al*, 2007). One clear distinction between sporadic and FAP tumours is the age of the host: all the sporadic adenomas in this study were from patients more than 56 years of age whereas FAP adenomas were collected from colonectomies performed on patients between 18 and 38 years of age. The impact of these age differences on rates of APC mutation and somatic hypermethylation is difficult to estimate: if methylation were purely stochastic, it would be reasonable to expect it to have a greater impact in older hosts, however in this study we have shown hypermethylation in tissue from a young population, which have been associated with the development of neoplasia. Interestingly 17% of the CpG sites in the younger FAP normal samples were fully methylated compared with 8% in sporadic and only 1% in unaffected normal samples (Figure 1B). It is possible that this is a result of contamination due to the difficulty in sampling ‘normal’ colonic mucosa from FAP patients but a recent report by Obrador-Hevia *et al* (2010) showed abnormal expression of *c-myc* and repression of *sFRP1* in macroscopically normal mucosa in patients with FAP, suggesting Wnt signalling pathway changes occur very early in FAP-associated tumourigenesis.

Most FAP adenomas showed qualitative changes that were similar to sporadic tumours. In those cases where *β*-catenin target genes were up-regulated, the degree of induction was comparable to that observed in sporadic adenomas, but the degree of *sFRP1* suppression in FAP adenomas was smaller than in sporadic cases. This could be the result of pre-existing change in the matched normal mucosa, but the raw Δ*C*_t_ data (not shown) do not indicate this. Although *sFRP1* gene hypermethylation is normally associated with complete silencing in sporadic adenomas, the lower levels of methylation observed in FAP adenomas appear to reflect partial suppression of gene expression. The outcome of this, or whatever other mechanisms are involved, is that FAP adenomas show significant reductions in sFRP1 transcription compared with matched normal mucosa, despite their elevated *β*-catenin activity. If Wnt ligand signalling is required for adenoma formation, it may be sufficient for the adenoma to limit expression of secreted Wnt antagonists to a level at or below that found in the normal mucosa: although the dominant mechanism for this in sporadic disease is complete silencing, the strong genetic drive of FAP selects for partial suppression. This is likely to be modulated by the distribution of histone marks around the locus (McGarvey *et al*, 2008) and will require examination in a separate study.

Investigating the outcome of *β*-catenin-independent Wnt signalling in these tumours could show whether similar outcomes are achieved in sporadic and FAP adenomas. No unambiguous markers of these signals have yet been identified in colorectal tumours but a possible response is phosphorylation of c-jun, which can be the result of *β*-catenin-independent Wnt signalling in other systems. We have previously reported a correlation between *sFRP1* silencing, *NKD1* expression and c-jun phosphorylation (Caldwell *et al*, 2008). In this study, we again found widespread phosphorylation (and nuclear localisation) of c-jun in FAP adenomas and in the cases that showed the highest levels of *sFRP1* suppression. FAP adenomas have increased cytoplasmic *β*-catenin compared with surrounding normal tissue but lack nuclear *β*-catenin (Bläker *et al*, 2003; Phelps *et al*, 2009; Anderson *et al*, 2002; this study). Phelps *et al* (2009) suggest a model wherein APC loss alone stabilises the levels of cytoplasmic *β*-catenin and that subsequent stabilisation and nuclear accumulation of *β*-catenin occurs following other secondary events, one of which may be c-jun phosphorylation. did not detect phospho-c-Jun (an indicator of JNK activity) in early adenomatous tissue from FAP patients. This contrasts with our findings where 9 out of 11 FAP adenomas stained positive for nuclear phospho-c-jun. Adenomas from these 11 FAP patients were available for mRNA expression analysis and showed elevated levels of the *β*-catenin target gene *NKD1* indicating increased *β*-catenin-activated transcription.

Cowling *et al* (2007) showed that *c-myc* expression led to a degree of *sFRP1* repression *in vitro*. If this were the case in colorectal tumourigenesis, we might expect a negative correlation between *sFRP1* mRNA and *c-myc* mRNA in this study. Pearson's correlation analysis of the FAP cohort shows a significant correlation coefficient of 0.78 between *sFRP1* down-regulation and *NKD1* up-regulation, but there is little correlation between *sFRP1* and *c-myc* levels (*r*=0.098), indicating that *c-myc* does not have a major role in suppression of *sFRP1* in these adenomas. Interestingly, Pearson's correlation analysis showed a significant positive correlation coefficient of 0.96 between *c-myc* and *FZD3* up-regulation in the FAP series, suggesting that these genes are under similar transcriptional control, which may be the result of Wnt signalling rather than simply *β*-catenin stabilisation (Caldwell *et al*, 2008).

We know that not all adenomas will become cancers and it may be that the molecular heterogeneity we observe in FAP adenomas represents the distinction between adenomas that are more or less likely to become invasive tumours. It is equally possible that, as FAP patients have a large number of adenomas, the heterogeneity represents different stages of adenoma development. The results of our studies however support *sFRP1* suppression being an early event in tumourigenesis, as it occurs even in FAP adenomas of 3 mm or less.

We have shown that most FAP adenomas share the changes in expression of Wnt pathway components that occur in sporadic disease but show a greater degree of heterogeneity, even within a single individual. Although the significance of this is as yet unclear, it may well have an impact on the sensitivity of a subset of tumours to targeted chemopreventive agents. These features could be characterised prospectively in resistant tumours in clinical trials of novel agents.

## Figures and Tables

**Table 1:** Summary of the clinical features of the three sample groups (normal unaffected, sporadic disease and FAP patients)

**Sample group**	**Sex (M/F)**	**Median age in years (range)**	**Total biopsy no.**	**Site of biopsy – right/left side of colon (ND)**	**Median size of adenoma in mm (range)**	**Pathology (TA/TVA)**	**Grade of dysplasia (low/high)**
Normal	6/1	60 (35–68)	12 control	1/11	—	—	—
Unaffected			—	—	—	—	—
Sporadic	12/10	65 (56–79)	22 control	0/22	—	—	—
Disease			22 adenoma	1/21	10 (5–30)	10/12	19/3
FAP patients	9/9	24 (18–38)	18 control	9/7 (6)	—	—	—
			30 adenoma	9/15 (6)	5 (2–35)	29/1	29/1

Abbreviations: M=male; F=female; TA=tubular adenoma; ND=not documented; TVA=tubular villus adenoma.

Right-sided adenomas are taken between the rectum and splenic flexure, left-sided adenomas are from the transverse colon to the caecum.

**Table 2:** Detailed information on the FAP adenoma samples used in the methylation and expression analyses

**FAP adenoma**	**Expr**	**Meth**	**Sex M/F**	**Age in years**	**Site of biopsy – right/left side of colon (ND)**	**Size of adenoma (mm)**	**Pathology** **(TA/TVA)**	**Grade of dysplasia (low/high)**
1a	Y	Y	F	32	L	2	TA	Low
1b	Y	Y			L	2	TA	Low
1c	Y	Y			L	2	TA	Low
1d	Y	Y			L	2	TA	Low
2a	Y	N	F	29	R	5	TA	Low
2b	Y	N			L	5	TA	Low
2c	Y	N			L	5	TA	Low
3a	Y	Y	M	20	L	5	TA	Low
3b	Y	Y			R	5	TVA	High
3c	Y	Y			L	35	TA	Low
4a	Y	N	F	26	R	6	TA	Low
4b	Y	N			L	9	TA	Low
5a	Y	Y	F	26	R	3	TA	Low
5b	Y	Y			R	3	TA	Low
6a	Y	N	M	21	ND	2	TA	Low
7a	Y	Y	F	23	ND	5	TA	Low
8a	Y	Y	M	20	ND	3	TA	Low
9a	Y	Y	M	19	R	5	TA	Low
10a	Y	Y	M	19	ND	3	TA	Low
11a	Y	Y	F	36	R	2	TA	Low
11b	Y	Y			R	2	TA	Low
11c	Y	Y			L	2	TA	Low
11d	Y	Y			L	3	TA	Low
12a	Y	Y	M	38	L	2	TA	Low
13a	Y	Y	F	27	ND	5	TA	Low
14a	Y	Y	F	35	L	10	TA	Low
15	N	Y	F	25	ND	5	TA	Low
16	N	Y	M	18	L	10	TA	Low
17	N	Y	M	18	R	5	TA	Low
18	N	Y	M	19	L	7	TA	Low

Abbreviations: ND=not documented; FAP=familial adenomatous polyposis; Expr=mRNA expression analysis performed; Meth=methylation analysis performed; M=male; F=female; TA=tubular adenoma; TVA=tubular villus adenoma.

**Table 3:** Sequences of primers used in real-time RT-PCR analysis

	**Assay type**	**Primers**
*Human PCR assays*		
*sFRP1* (NM_003012)	TaqMan	F, 5′-CCAATCCCACCGAAGCCT-3′
		R, 5′-ATGATGGCCTCAGATTTCAACTC-3′
		P, 5′-CAAGCCCCAAGGCACAACGGTG-3′
*NKD1* (NM_033119)	TaqMan	F, 5′-TCGCCGGGATAGAAAACTACA-3′
		R, 5′-CAGTTCTGACTTCTGGGCCAC-3′
		P, 5′-CCAATTTGGGCCTGGCTCCCC-3′
*FZD3* (NM_017412)	TaqMan	F, 5′-CATGGAGATGTTTGGTGTTCCTT-3′
		R, 5′-AAGTCGAGGATATGGCTCATCAC-3′
		P, 5′-TCTGGGAACCTACTGCATTCCATATCTTCAGG-3′
*c-myc* (NM_002467)	TaqMan	F, 5′-AGCTGCTTAGACGCTGGATTTT-3′
		R, 5′-TTCCTGTTGGTGAAGCTAACGTT-3′
		P, 5′-CAGCCTCCCGCGACGATGC-3′
*KRT8* (NM_002273)	TaqMan	F, 5′-GATCGCCACCTACAGGAAGCT-3′
		R, 5′-ACTCATGTTCTGCATCCCAGACT-3′
		P, 5′-CCGGCTCTCCTCGCCCTCCA-3′

**Table 4:** *P*-values (Wilcoxon's rank-sum test) of methylation at each of the seven CpG sites tested in *sFRP1* in adenoma *vs* matched normal mucosa in both FAP and sporadic patients

**CpG site**	**FAP (*n*=14)**	**Sporadic (*n*=19)**
1	0.217	0.007
2	0.367	0.006
3	0.075	0.007
4	0.096	0.016
5	0.451	0.005
6	0.975	0.016
7	0.221	0.015
Average of site 3 and 4	0.030	0.010
Average of site 1 to 7	0.119	0.003

Abbreviation: FAP=familial adenomatous polyposis.
